# Efficiency of Treatment Plant and Drinking Water Quality Assessment from Source to Household, Gondar City, Northwest Ethiopia

**DOI:** 10.1155/2021/9974064

**Published:** 2021-05-29

**Authors:** Belay Desye, Biniam Belete, Zinabu Asfaw Gebrezgi, Tsegaye Terefe Reda

**Affiliations:** ^1^Department of Public Health, College of Medicine and Health Sciences, Adigrat University, Adigrat, Ethiopia; ^2^Department of Public Health, College of Health Sciences, Arsi University, Asella, Ethiopia; ^3^Public Health Emergency Management Office, Amhara, Ethiopia

## Abstract

**Introduction:**

Access to safe drinking water is essential to health, and it is a basic human right. However, drinking water treatment plant efficiency and its water quality are not well investigated in low-income countries including Ethiopia.

**Methods:**

A laboratory-based cross-sectional study was conducted among 75 water samples. Data analysis was carried out using SPSS version 22 to generate descriptive statistics, and one-way ANOVA was used to test statistically significant difference.

**Results:**

Physicochemical qualities of the water samples from tap water sources were found to be pH (6.88 ± 0.05), turbidity (5.15 ± 0.006 NTU), electrical conductivity (170.6 ± 0.1 *μ*S/cm), residual chlorine (0.19 ± 0.003 mg/L), and fluoride (1.17 ± 0.009 mg/L). The removal efficiency of turbidity, total hardness, and nitrate was found to be 94.4%, 52.3%, and 88.7%, respectively. Removal efficiency of the treatment plant for total coliforms up to 91.6% (15 ± 0.26 CFU/100 mL in tap water) and faecal coliforms up to 99% (1.51 ± 0.03 CFU/100 mL in tap water) was recorded. Parameters of pH, temperature, and faecal coliform were statistically significant different at *p* < 0.05 in tap water source. The overall efficiency of the treatment plant (68.5%) and the water quality index (76) were recorded.

**Conclusion:**

Based on the results, some of the investigated parameters of water quality (turbidity, residual chlorine, total coliform, and faecal coliform) were found to be not within the permissible limits of WHO guideline values for drinking water quality. The water quality index of the water samples was categorized under good water quality. To adequately treat drinking water and improve the treatment plant, adequate preliminary treatments like screening to reduce the incoming organic loading, proper chlorination of the drinking water system, and frequent monitoring and maintenance of the treatment plant system are required.

## 1. Introduction

Drinking water is indispensable for human existence. It is one of the most important of all-natural resources that exist on Earth [1]. Access to safe drinking water is essential to health, a fundamental human right, and a component of effective policy for health maintenance. Every effort should be made to improve and address safe drinking water for better health [2, 3]. Drinking water quality should be completely free from any pathogenic microorganisms and physicochemical contaminants that can cause health impact [3, 4].

Water treatment involves removing of all impurities that are potentially harmful in water supply for human consumptions [5]. Improving the quality of water before used by consumers is depending on the efficiency of drinking water treatment processes, which must be safe and meet the standard criteria for public health [6, 7]. Poor drinking water quality is significantly affecting the health of consumers. Poor sanitation and contaminated drinking water are linked to transmission of disease such as diarrhea, cholera, dysentery, and polio [3, 8].

Water quality is a growing global concern and over 80% of the human diseases in the world are caused by unsafe water source and inadequate environmental sanitation practices [3]. Globally, 2 billion people drink water that is faecally contaminated and 4.5 billion people used unimproved sanitation system. Over half of these peoples live in sub-Saharan Africa [8]. Most of population live in low-income countries suffers from health problems associated with lack of drinking water and contaminated water sources with microorganism [9]. Nowadays, water sources are being polluted largely by agricultural practice and industrial chemical waste disposals due to cross contamination with sewerage, illegal connections, leakages, and corrosions [3, 10].

Although there was some routine quality assessment in tap water sources of major urban towns of Ethiopia, little attention is being given to drinking water quality issues and quantity by water supply agencies. In the country, there is a lack of drinking water quality surveillance and monitoring programmes [10, 11]. Despite the alarming rate of water pollution and the huge disease burden associated with unsafe water supply, very little effort has been put to investigate the efficiency of existing water treatment systems and drinking water quality in low-income countries. Any such limited study was conducted in the other cities of the country, but for the case of Gondar city nothing is done so far. Therefore, this study may have its own contribution and implication for water supply agencies and policy and decision makers to take corrective action. Hence, the aim of this study is to evaluate treatment plant efficiency and drinking water quality assessment from source to household in Gondar city, Northwest Ethiopia.

## 2. Materials and Methods

### 2.1. Study Area

The study was conducted in Gondar city, Amhara regional state, which is located 739 km northwest of Addis Ababa, the capital city of Ethiopia. The city of Gondar found at latitude and longitude of 12°36'N 37°28'E with an elevation of 2133 m above sea level. The annual mean temperature of the area is 20.5°C (17.2–23.9°C) and annual rainfall is about 1000 mm (600–1400 mm). The total population of Gondar was 207,044, of whom 98,120 were men and 108,924 were women according to the national census conducted by the Central Statistical Agency (CSA) [12].

Angereb water treatment plant is a major source of drinking water to Gondar city. It is about 2.3 km in the east direction of the center of the city. The source of the treatment plant was mainly from surface water (84%) and with additional eight boreholes. The final treated water was lifted via high-lift pumps to a reservoir situated in Debre Birihan Selassie for onward distribution to the city administration. The water distribution system of the city contains service reservoirs, distribution pipes, and pressure break tanks at different locations.

### 2.2. Study Design, Period, and Sample Size Determination

A laboratory based cross-sectional study was conducted in Gondar city, Northwest Ethiopia, from June 30 to August 30, 2020. A total of 75 water samples were collected from inlet, outlet, main distribution site, and household taps using WHO recommended minimum sample numbers for piped drinking water [13].

### 2.3. Experimental Procedure

#### 2.3.1. Water Sample Collection and Analysis

Grab samples were collected from inlet of treatment plant (before undergoing any treatment), outlet of treatment plant (when it leaves the disinfection point), main distribution tank, and household taps in the city. Three times from each sampling sites were collected.

Water samples were collected aseptically using 300-milliliter (mL) sterile glass bottles for bacteriological analysis and a sterile one-liter polyethylene (PET) bottles for physicochemical analysis. Prior to sampling, the glass bottles were sterilized using an autoclaved for 15–20 minutes at 120°C to avoid contamination and the PET bottles were washed and rinsed with distilled water. For chlorinated water (treated water), sample collection was carried out using glass bottles that are autoclaved with 0.1 mL 3% sodium thiosulphate (Na_2_S_2_O_3_) to neutralize the chlorine present in the sample. The tap was turned on at maximum flow rate, and the water was allowed to flow for 2 minutes. The tap was disinfected for a minute using a 70% alcohol and allowed to flow at a medium rate for 2 minutes. Previously sterilized glass and clean PET bottles were open for collecting water samples by holding the bottle steady under the water jet, while the cover cup was held in up-down position. Samples at treatment plant units were taken according to surface water sampling procedure by dipping the sampling container to 20 cm of the water body [3]. Samples were sealed, labeled, and transported in icebox (4°C) to the Microbiology Laboratory of the University of Gondar within 6 hours of sample collection. The sampling protocol was carried out scrupulously following the standard methods of American Public Health Association (APHA) [14].

#### 2.3.2. Bacteriological Analysis

Total coliform (TC) and faecal coliform (FC) were determined using membrane filtration (MF) technique. 100-milliliter sample was filtered through 0.45 *μ*m membrane filter, under vacuum. The membrane filter was then layered to 2 mL prepared membrane lauryl sulfate broth with absorbent pad and incubated at 37°C for 48 hours and 44 ± 0.5°C for 24 hours, respectively. After incubation period, the plates were examined for the development of yellow colonies, and it was expressed in colony forming unit (CFU) per 100 mL of water sample [14]:

(1) $$\begin{array}{c} \frac{\text{number of colonies}}{100\text{ mL}}=\frac{\text{coliform colonies counted}}{\text{mL sample filtered}}\times 100. \end{array}$$

#### 2.3.3. Physicochemical Analysis

Physical parameters such as pH, temperature, electrical conductivity (EC), dissolved oxygen (DO), turbidity, and total dissolved solid (TDS) were measured in situ by pretested and calibrated portable digital multiparameter probe (HACH), whereas total hardness, residual chlorine (RC), total alkalinity, nitrate, iron, manganese, fluoride, sulphate, and phosphate were determined using DR 2800 spectrophotometer (Loveland, USA).

### 2.4. Water Quality Index (WQI)

Water quality index is a useful and effective way to determine the overall quality of water. It is also a very useful average tool used to convert large number of water quality data into a single cumulatively derived number. The WQI model gives information about the overall quality of water to the policy makers and concerned body [15, 16]. The weight numbers of each parameters for calculating WQI were assigned based on their perceived effects on human health and significance on drinking purpose. The WQI can be computed with parameters such as the relative weight (*Wi*), the quality rating (*qi*), and parameter subindex (*Si*). The WQI value can be obtained from the following equation:

(2) $$\begin{array}{c} Wi=\frac{wi}{\sum wi},\\ qi=\frac{Ci}{Si}\times 100,\\ \text{SI}i=Wiqi,\\ \text{WQI}=\sum \text{SI}i, \end{array}$$

whereas *Wi* is the relative weight, *wi* is the weight of each parameter and ∑*wi* is the sum of all the parameters, *qi* is the quality rating scale, *Ci* is the concentration of each parameter in each water sample in mg/L, and *Si* is the World Health Organization (WHO) standard for each parameter in milligrams per liter. The computed WQI values are classified into five categories as defined [17] in Table 1.

### 2.5. Data Quality Assurance

To maintain the quality of the data, proper sterilization of equipment's, aseptic procedures, control media, blank measurements, and triplicate analysis were used. In addition, standard methods of sampling techniques and analysis procedures were used.

### 2.6. Data Management and Analysis

All the raw data was coded and entered to Microsoft Excel 2016 spreadsheet. Then, the data was exported to SPSS (version 24) for statistical analysis. Mean values and standard deviations were calculated. One-Way Analysis of Variance (ANOVA) was used to test statistically significant difference in tested parameters among sample sources. The overall efficiency of the treatment plant was calculated using the following formula:

(3) $$\begin{array}{c} \text{removal efficiency }\left(\%\right)=\left(\frac{Ci-Ce}{Ci}\right)\times 100, \end{array}$$

whereas *Ci* is the inlet concentration and *Ce* is the effluent concentration of pollutants.

## 3. Results

### 3.1. Characterization of Physicochemical and Bacteriological Quality of Water

The characteristics of physicochemical parameters in tap water source were pH (6.88 ± 0.05), turbidity (5.15 ± 0.006 NTU), EC (170.6 ± 0.1 *µ*S/cm), RC (0.19 ± 0.003 mg/L), fluoride (1.17 ± 0.009), total hardness (62.7 ± 0.5 mg/L), and nitrate (3.84 ± 0.005 mg/L), whereas bacteriological parameters in tap water source TC (15 ± 0.26 CFU/100 mL) and FC (1.51 ± 0.03 CFU/100 mL) were recorded. Parameters of pH and FC were statistically significant different at *p* < 0.05 in tap water source as depicted in Table 2.

### 3.2. Drinking Water Treatment Plant Efficiency

Removal efficiency of the treatment plant for physicochemical parameters turbidity (94.4%), total hardness (52.5%), and nitrate (88.7%) were recorded, whereas removal efficiency for bacteriological parameters TC (91.6%) and FC (99%) was found. Fluoride, sulphate, and iron were not statistically significantly different at *p* < 0.05 in removal efficiency as depicted in Table 3.

### 3.3. Water Quality Index of Drinking Water

Parameters like pH, DO, EC, total hardness, iron, and fluoride are in line with the permissible limits of WHO. However, turbidity and nitrate were not within the permissible limits of WHO. The WQI was 76 as depicted in Table 4.

## 4. Discussion

### 4.1. Physicochemical Quality of Water

Physicochemical contaminants have been proved to cause adverse health effects in humans because of prolonged exposure through drinking water. These include both organic and inorganic chemicals. Some of them are toxic to humans and affect the aesthetic quality of water [13].

Average pH records of tap water samples (6.88 ± 0.05) were found within the permissible range of WHO drinking water guideline value (6.5–8.5) [3]. Statistically significant difference was observed among the water samples collected from different sampling points (*p* < 0.05). The pH values in tap water obtained in this study are lower than the results of previous studies (7.12) at Jimma town [11] and (7.6) at Akaki Kality subcity, Addis Ababa [10]. The variation might be due to natural geological conditions and minerals found in the local rock of the water sources [18]. The lowest pH value in this study was 6.24 in main distribution. The possible reason for this might be the use of chemical alum to coagulation process, in treatment plant as reported [19]. The addition of excess aluminum sulfate (Al_2_(SO_4_)_3_) would reduce the pH of the water. The pH values less than 6.5 enhance corrosion in water pipes, while pH values greater than 8.5 are not suitable for effective disinfection [3].

The mean value of turbidity was found (5.15 ± 0.006 NTU) in tap water as depicted in Table 2. There was statistically significant difference (*p* < 0.05) among mean turbidity of different water sampling points except tap water sources. The finding of turbidity was consistent with the study reported at Jimma town, Ethiopia [11]. However, it was higher than the previous studies conducted in Ethiopia [20–22]. The variation might be due to the nature of raw wastewater, the sampling period, and the local runoff conditions [3, 11, 22]. The treatment plant efficiency for turbidity removal was found (94.4%), which is consistent with (94.8%) a study conducted in Jimma town, Ethiopia [11]. For effective disinfection in drinking water treatment plant, turbidity of 0.5 NTU is recommended. However, according to the WHO, up to 5 NTU is considered acceptable for drinking water treatment [3]. However, the turbidity value of tap water (5.15 NTU) in this study did not comply with WHO standard (<5 NTU). This might be due to the fact that the sampling period was in rainy season that can be crossly contaminated with runoffs [10, 11]. Water with high turbidity affects treatment plant system with reduction of the bacteriological removal and chemical consumption since high levels of turbidity may make it difficult to be disinfected appropriately [23].

Electrical conductivity in natural water is the measure of the water's ability to conduct electric current [24]. Dissolved salts such as potassium chloride and sodium chloride mainly affect electrical conductivities [25]. Electrical conductivities among sampled points were varied from (153–170.6 *μ*S/cm). The lowest EC (153 *μ*S/cm) was recorded from untreated water sources, while the higher EC was recorded from water samples obtained after disinfection points (168 *μ*S/cm) in main distribution and (170.6 *μ*S/cm) in tap waters. There was statistically significant difference (*p* < 0.05) among mean EC in water samples at the source point. The increments of EC might be attributed to the addition of chemicals for coagulation and disinfection for removal of turbidity [26, 27]. However, EC of water sources in this study was found below the WHO drinking water permissible limit of (1000 *μ*S/cm) [3]. The average EC of tap water sources (170.6 *μ*S/cm) in this study was lower than a study conducted in Jimma zone, Ethiopia (366.93 *μ*S/cm) [28].The variation might be due to the local geological conditions, soil types, and agricultural activities of the study area [3, 29].

Chlorine is added to water for the purpose of killing disease causing bacterial pathogens to prevent the spread of waterborne disease. For attaining this purpose, chlorine is added during water treatment process to untreated water (initial chlorine) and treated water (final chlorine). Chlorine in treated water is measured to make sure that they are sufficiently high to remove pathogenic bacteria and maintained the safety for drinking water in distribution system which travel for long distance to reach consumers' taps [30, 31]. The RC of the water samples from source point, disinfection point, main distribution, and tap water were 2.25 ± 0.16 mg/L, 2.95 ± 0.49 mg/L, 3.12 ± 0.6 mg/L, and 0.19 ± 0.003 mg/L, respectively, as depicted in Table 2. The findings were supported by WHO; the higher level of RC should be close to the disinfection point and the lower level at the far extremities of the supply networks [3]. The RC content of water samples in tap water (0.19 mg/L) in this study was slightly lower than (0.21 mg/L) at Nekemte Oromia, Ethiopia [22]. However, the finding of RC in tap water sources was found not within the permissible limit of WHO (0.2–0.5 mg/L) [3]. The possible justification for reduced level of RC at the tap water source could be cross contamination via distribution lines [3, 11, 22].

The total hardness of the water samples from source point, disinfection point, main distribution, and tap water was 310.7 ± 9.02 mg/L, 147.7 ± 2.5 mg/L, 137.7 ± 11.2 mg/L, and 62.7 ± 0.5 mg/L, respectively, as depicted in Table 2. The finding of total hardness in tap water (62.7 mg/L) was relatively higher than that of a study (41.18 mg/L) conducted in Jimma town, Ethiopia [11]. This might be due to the source of water and natural geological conditions [3]. There was statistically significant difference (*p* < 0.05) among mean total hardness of different water sampling points except tap water sources. The total hardness value in tap water source in this study was below the permissible limit of WHO (300 mg/L) [3]. The degree of hardness of drinking water is important for aesthetic acceptability by consumers, for economic and operational consideration [3].

The fluoride amounts in water samples from source point, disinfection point, main distribution, and tap water were 2.7 ± 0.21 mg/L, 2.07 ± 0.25 mg/L, 1.85 ± 0.05 mg/L, and 1.17 ± 0.009 mg/L, respectively, as depicted in Table 2. The fluoride amount in tape water source was found below the permissible limit of WHO guideline values (1.5 mg/L) [3]. The finding of fluoride amount in tap water source in this study was higher than the range value 0.18–0.65 mg/L found in East Azerbaijan, Iran [32], and 0.15 mg/L in Jimma town, Ethiopia [11]. The variation might be due to the source of raw wastewater and the treatment capability of the treatment plant [3, 11]. In some of the ground water sources in India, Africa, and the rest of the world, an excess amount of fluoride (up to 100 m/L) has also been reported [33].

The measured nitrate concentration of the water samples from source point, disinfection points, main distribution, and tap waters was 76.7 ± 7.64 mg/L, 8.7 ± 3.5 mg/L, 4.7 ± 1.5 mg/L, and 3.84 ± 0.05 mg/L, respectively, as depicted in Table 2. The nitrate content in untreated water sources was significantly higher than the rest sampling points. However, there was no statistically significant difference (*p* < 0.05) in main distribution and tap water. Nevertheless, the nitrate level of the water sources in tap water was much less than the permissible limit of WHO for drinking water quality (<50 mg/L). This indicates that the nitrate concentration is not the problem of water in the study area. The nitrate level of the tap water sources (3.84 mg/L) measured in this study is higher than 0.89 mg/L in Jimma town, Ethiopia [11] and 3.6 mg/L in Nekemte Oromia, Ethiopia [22]. The variation might be due to the source of untreated water, the local runoff, and sewage condition [3].

The study revealed that the concentrations of major physicochemical parameters analyzed are within the permissible limits of the WHO drinking water guidelines with the exception of turbidity and residual chlorine.

### 4.2. Bacteriological Quality of Water

In developing countries like Ethiopia, majority of the population is not adequately supplied with drinking water from protected and managed water supply network and are forced to use unprotected water that may be unsafe for domestic purpose as a result of contamination through natural and anthropogenic sources [3]. Bacteriological quality is the primary issue in any water quality assessment program, especially those used for domestic purposes. Indicator microorganisms are identified to demonstrate the presence of human and animal wastes and hence the potential presence of pathogens in drinking water [34, 35].

In the present study, total coliforms in the water sampling points were varied from 10.13 to 120.2 CFU/100 mL with removal efficiency of 91.6%, whereas FC in the water samples was varied from 0.49 to 50.2 CFU/100 mL) with the removal efficiency of 99%. Although the treatment plant significantly reduced the number of TC and FC, the water samples contain a large number of bacteria. Similarly, a previous study had detected TC and FC in tap water samples in Addis Ababa [10], Nekemte Oromia [22], and Jimma town, Ethiopia [11]. The reduction of the TC and FC counts in the household tap water samples could be attributed to free RC applied at the disinfection point of the treatment system [13]. Both TC and FC were not within the permissible limits of WHO drinking water guideline values (0/100 mL) [3]. The presence of FC in drinking water may affect human health in many ways, and it is an indicator of the presence of pathogenic bacteria [36]. This might be due to leaching during rainy season, high runoff, and inadequate of the treatment plant. The treatment process during the rainy season could be affected by turbidity of the surface water sources due to high rainfall [37]. In addition, lack of regular water quality monitoring in the water treatment plant and distribution system may be a possible explanation for the presence of bacteriological in tap water samples [10, 11]. Results analysis using one-way ANOVA test revealed a statistically significant difference of FC counts among main distribution and tap water source samples (*p* < 0.05).

Moreover, the presence of bacteriological parameters in tap water sources might be due to the fact the treatment plant is far away from the city. Hence, the interconnection between the site of the treatment plant and the tap up to the household of the consumers may accumulate pathogenic microorganisms by formation of biofilms. It is therefore highly recommended that the water sources be treated or boiled at household level before it is used for domestic purposes [38, 39].

### 4.3. Water Quality Index of Water Samples

Water quality index was calculated in order to describe the overall quality status of the drinking water. The computed water quality index was 76 as shown in Table 4, and it can be categorized under good water quality (but not excellent) as defined [17]. This might be due to inadequate treatment system, poor water quality monitoring, and contaminations at different interconnection [3]. The finding was consistent with a study conducted at Cairo, Egypt, which was categorized under good water quality [39]. However, the finding of WQI in this study was higher than a study conducted in Jimma town, Ethiopia, which was categorized under poor water quality [11]. This variation might be due to the nature of source of water, the design of the treatment plant, and the local runoff conditions [11, 39]. Generally, it can be said that the water was somewhat suitable for drinking according to the WQI. Much more attention should be paid to these parameters within not meeting the permissible limits of drinking water.

*Limitations of the Study.* This cross-sectional study did not show us the effect of seasonal variation for treatment plant efficiency and water quality assessment.

## 5. Conclusions

Based on the results, some of the investigated parameters of water quality (turbidity, RC, TC, and FC) were found to be not within the permissible limits of WHO guideline values for drinking water quality. The removal efficiency of the treatment plant was in a satisfactory level, and the water quality index was categorized under good water quality. To adequately treat drinking water and improve the treatment plant, it is required an adequate preliminary treatment like screening to reduce the incoming organic loading, proper chlorination of the drinking water system, and frequent monitoring and maintenance of the treatment plant system. Therefore, the treatment plant strategies should be developed from source to household taps to deliver safe water to reducing human health risks.

## Figures and Tables

**Table 1:** Water quality classifications.

WQI value	Rank
**<50**	Excellent water
**50–100**	Good water
**100–200**	Poor water
**200–300**	Very poor water
**>300**	Unsuitable for drinking

**Table 2:** Mean ± SD of physicochemical and bacteriological analysis of water samples at different sampling points in Gondar city, Northwest Ethiopia, June 30 to August 30, 2020.

Parameters	Source point	Disinfection point	Main distribution	Tap water
pH	7.17 ± 0.32^*∗*^	6.27 ± 0.15^*∗*^	6.24 ± 0.07^*∗*^	6.88 ± 0.05^*∗*^
Temperature (°C)	24 ± 1	20.33 ± 1.53^*∗*^	19.67 ± 2.08	25.3 ± 0.13^*∗*^
DO (mg/L)	15 ± 1	18.33 ± 1.53	19.17 ± 0.76	6.5 ± 0.04
Turbidity (NTU)	84.3 ± 6.03^*∗*^	4.5 ± 0.74^*∗*^	4.77 ± 0.65^*∗*^	5.15 ± 0.006
TDS (mg/L)	245.7 ± 10.7^*∗*^	62.33 ± 2.52	62.3 ± 2.08	50.7 ± 0.57
EC (*μ*S/cm)	153 ± 14.5^*∗*^	164.3 ± 5.13	168 ± 1.73	170.6 ± 0.1
RC (mg/L)	2.25 ± 0.16^*∗*^	2.95 ± 0.49^*∗*^	3.12 ± 0.6^*∗*^	0.19 ± 0.003
Fluoride (mg/L)	2.7 ± 0.21	2.07 ± 0.25	1.85 ± 0.05	1.17 ± 0.009
Total hardness (mg/L)	310.7 ± 9.02^*∗*^	147.7 ± 2.5^*∗*^	137.7 ± 11.2^*∗*^	62.7 ± 0.5
Total alkalinity (mg/L)	138.7 ± 8.5^*∗*^	75.7 ± 2.1^*∗*^	73 ± 1^*∗*^	32 ± 0.1
Nitrate (mg/L)	76.7 ± 7.64^*∗*^	8.7 ± 3.5^*∗*^	4.7 ± 1.5	3.84 ± 0.005
Sulphate (mg/L)	139.7 ± 5.5	65.3 ± 7.6^*∗*^	59 ± 3.6^*∗*^	1.2 ± 0.008
Phosphate (mg/L)	90.3 ± 4.5^*∗*^	42.8 ± 2.5^*∗*^	40.7 ± 1.5	1.73 ± 0.007
Iron (mg/L)	1.2 ± 0.0.3^*∗*^	0.5 ± 0.15	0.48 ± 0.025	0.15 ± 0.004
Manganese (mg/L)	2.3 ± 0.2^*∗*^	0.28 ± 0.2	0.48 ± 0.24^*∗*^	0.53 ± 0.011
TC (CFU/100 mL)	120.2 ± 0.76	10.13 ± 0.35	10.6 ± 0.1	15 ± 0.26
FC (CFU/100 mL)	50.2 ± 0.61	0.49 ± 0.02	0.6 ± 0.1^*∗*^	1.51 ± 0.03^*∗*^

^*∗*^The parameter was significant at *p* < 0.05. DO = dissolved oxygen, TDS = total dissolved solid, EC = electrical conductivity, RC = residual chlorine, TC = total coliform, and FC = faecal coliform.

**Table 3:** Mean ± SD of water treatment plant efficiency for selected parameters in Gondar city, Northwest Ethiopia, June 30 to August 30, 2020.

Parameters	Inlet	Outlet	Removal efficiency (%)
Turbidity (NTU)	84.3	4.68	94.4^*∗*^
TDS (mg/L)	245.7	62.3	74.6^*∗*^
Fluoride (mg/L)	2.7	2.07	23.3
Total hardness (mg/L)	310.7	147.7	52.5^*∗*^
Total alkalinity (mg/L)	138.7	75.7	45.4^*∗*^
Nitrate (mg/L)	76.7	8.7	88.7^*∗*^
Sulphate (mg/L)	139.7	65.3	53.3
Phosphate (mg/L)	90.3	42.77	52.6^*∗*^
Iron (mg/L)	1.2	0.5	58.33
Manganese (mg/L)	2.3	0.28	87.8^*∗*^
TC (CFU/100 mL)	120.2	10.1	91.6^*∗*^
FC (CFU/100 mL)	50.2	0.49	99^*∗*^
Average (%) efficiency	—	—	**68.5**

^*∗*^The parameter was significant in removal efficiency at *p* < 0.05. TDS = total dissolved solid, TC = total coliform, and FC = faecal coliform.

**Table 4:** Water quality index of selected physicochemical parameters of drinking water in Gondar city, Northwest Ethiopia, June 30 to August 30, 2020.

Parameters	WHO standard (*Si*)	*Ci*	*Wi*	*Wi*	*qi* (%)	SI*i*
pH	6.5–8.5	6.88	4	0.125	80.9	10.11
DO (mg/L)	>6	6.5	4	0.125	108.3	13.54
Turbidity (NTU)	5	5.1	5	0.1563	102	15.94
EC (*μ*S/cm)	1000	170.6	3	0.0934	17.06	1.6
Nitrate (mg/L)	50	50.7	5	0.1563	101.4	15.85
Total hardness (mg/L)	300	62.7	3	0.0934	20.9	1.95
Iron (mg/L)	0.3	0.15	3	0.0934	50	4.67
Fluoride (mg/L)	1.5	1.17	5	0.1563	78	12.19
—	—	—	∑*wi* = 32	—	—	WQI = 76

*Note*. *Ci* = concentration of each parameter, *wi* = weight of each parameter, *Wi* = relative weight, *qi* = quality rating scale, SI*i* = parameter subindex, DO = dissolved oxygen, and EC = electrical conductivity.

## Data Availability

The data used to support the findings of this study are available from the corresponding author upon request.
